# Supplementary material for: HSF1 phosphorylation establishes an active chromatin state via the TRRAP–TIP60 complex and promotes tumorigenesis
Source: Nat Commun. 2022 Jul 29;13:4355. doi: 10.1038/s41467-022-32034-4 (PMC9338313; doi:10.1038/s41467-022-32034-4)
Supplement: Supplementary file 1 — Supplementary Information [file 41467_2022_32034_MOESM1_ESM.pdf]

Supplementary Information

HSF1 phosphorylation establishes an active chromatin state via the TRRAP-TIP60 complex and promotes tumorigenesis

Mitsuaki Fujimoto, Ryosuke Takii, Masaki Matsumoto, Mariko Okada, Keiich I. Nakayama, Ryuichiro Nakato, Katsunori Fujiki, Katsuhiko Shirahige, and Akira Nakai1, *

*To whom correspondence should be addressed. E-mail: anakai@yamaguchi-u.ac.jp

Table of contents:

Supplementary Figures

Supplementary Fig. 1

Supplementary Fig. 2

Supplementary Fig. 3

Supplementary Fig. 4

Supplementary Fig. 5

Supplementary Fig. 6

Supplementary Tables

Supplementary Table 1

Supplementary Table 2

Supplementary Table 3

Supplementary Table 4

Supplementary References

a

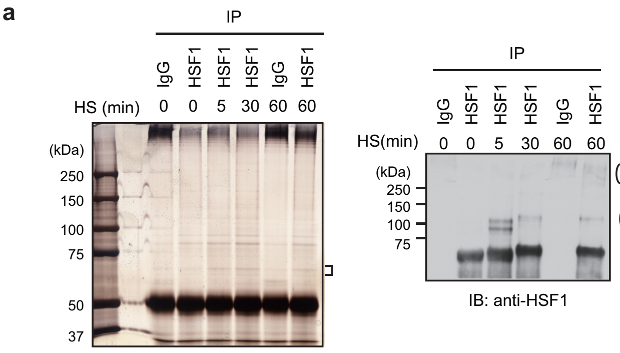

c

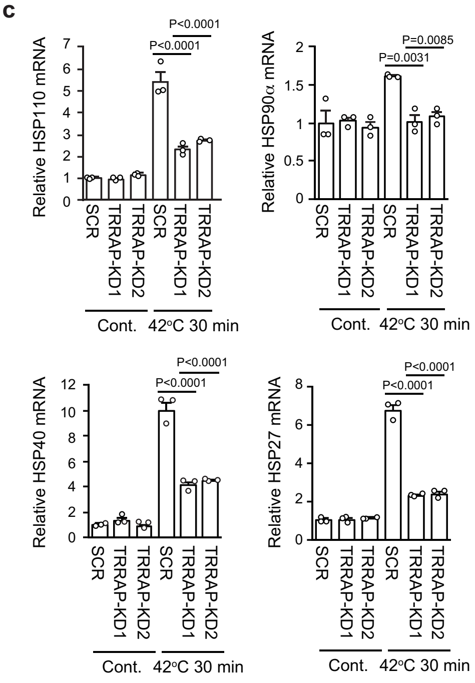

b

Gene name	Peptides 0 min	Peptides 5 min	Peptides 30 min	Peptides 60 min
PSMC1	0	2	4	2
SMCHD1	0	3	5	4
KPNA2	0	1	5	2
EP300	0	1	5	6
TRRAP	0	1	12	6
IPO7	0	2	2	2
NUP107	0	2	3	2
NSF	0	2	3	2
PDCD6IP	0	1	2	2
EIF4G1	0	0	7	2
SRSF	0	0	4	2
BCL9L	0	0	2	2
CREBBP	0	0	4	5
NOP2	0	0	5	2
PLK1	0	0	2	2
NOP56	0	0	1	2
MOV10	0	0	2	2
NAT10	0	0	2	4
EIF3M	0	0	3	4
ASCL3	0	0	2	2
MORC2	0	0	1	2
TRIM24	3	9	18	13
DYNC1H1	3	6	26	5
TRIM29	2	4	5	4
CPS1	1	3	5	4
RUVBL1	1	3	4	3
KPNA1	1	2	3	3
SEC31A	3	3	9	6
TUBB2C	6	5	14	11
EP400	1	1	6	3
ACBD3	1	1	2	3
SMC4	1	1	7	3
CCT6A	2	9	10	5
CPSF7	2	4	5	4
YARS	2	3	5	4
TRIM33	3	9	19	16
CCT7	7	13	15	9
CCT5	4	7	10	7
EEF1G	5	7	9	7
PTBP1	4	8	11	8
HRNR	9	10	17	18
KARS	2	4	6	7
EPRS	4	9	13	7
NONO	10	14	24	16
FASN	40	36	59	44

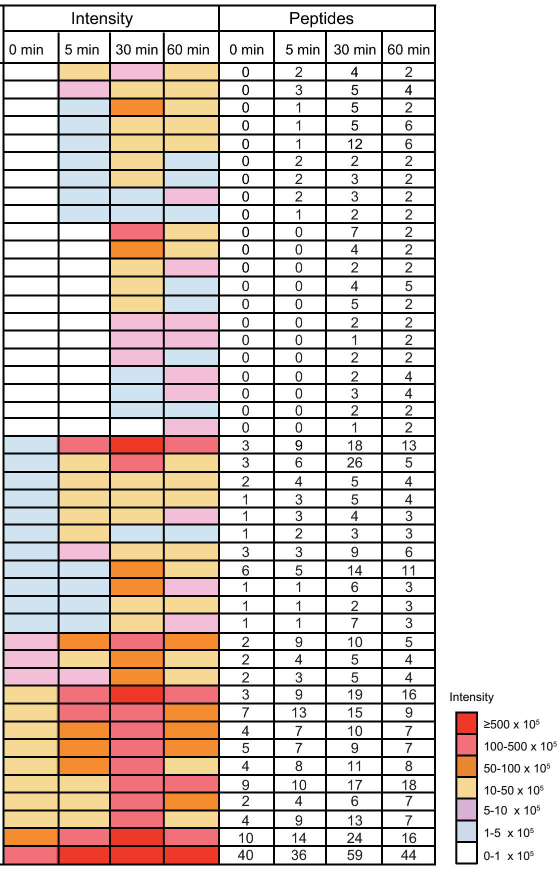

d

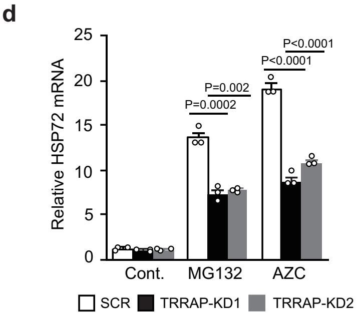

e

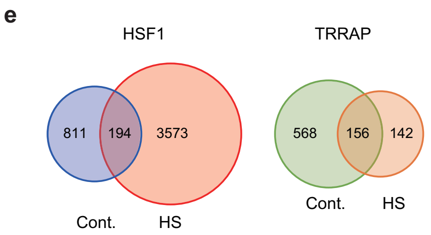

f

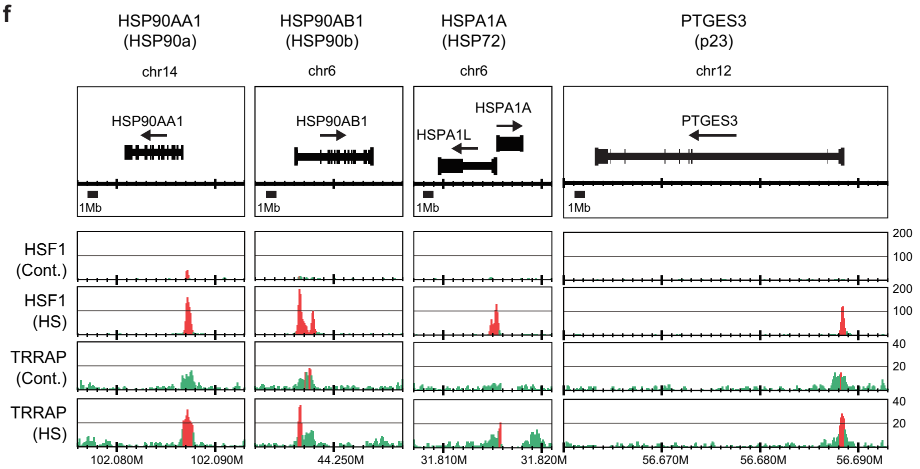

Supplementary Fig. 1. Identification and characterization of HSF1-interacting proteins.

a Silver staining and immunoblotting of elutions from HSF1 ChIP preparations. HeLa cells were treated with heat shock at 42°C for the indicated periods (HS). Proteins in HSF1 or IgG ChIP preparations were resolved on 8% SDS-PAGE and visualized by silver staining (left). Expected positions of HSF1 monomer bands are indicated on the right. Alternatively, the proteins were subjected to HSF1 immunoblotting (right). Positions of HSF1 monomer, dimer, and trimer are indicated.

b Time-dependent abundance profile of proteins in HSF1 ChIP preparations. HSF1-interacting proteins were identified by ChIP-MS (Supplementary Data 2). LFQ intensity value and identified peptide numbers at different time points are shown. Differences of the identified peptide numbers in HSF1 ChIP preparations from untreated or treated with heat shock for 60 min were calculated, and 45 proteins highly enriched upon heat shock (difference of peptide numbers > 2) are listed. Proteins related to histone modifications and protein kinases are indicated in red and blue, respectively.

c TRRAP KD reduces the mRNA expression of major HSPs. TRRAP KD cells were treated with heat shock. mRNA levels of HSP110 (HSPH1), HSP90α (HSP90AA1), HSP40 (DNAJB1), and HSP27 (HSPB1) were quantified and the levels relative to that in control SCR-treated cells (fold induction) are shown.

d TRRAP KD reduces the expression of HSP72 mRNA during treatment with proteotoxic inducers. TRRAP KD cells were treated with 10 μM MG132 for 6 h or 5 mM AZC for 6 h. mRNA levels of HSP72 (HSPA1A) were quantified and fold inductions are shown.

e Venn diagram of HSF1 and TRRAP ChIP-seq binding peaks in control (Cont.) and heat shocked (HS) cells. Numbers of binding peaks are indicated.

f ChIP-seq binding profiles for HSF1 and TRRAP at *HSP90AA1*, *HSP90AB1*, *HSPA1A*, and *PTGES3* loci. Normalized read numbers are shown. Significantly enriched regions identified as peaks are indicated in red. Arrows indicate the 5'-to-3' orientation of each gene.

Norminal *p*-values were determined by one-way ANOVA, followed by Tukey-Kramer test in c and **d**. Error bars indicate SEM (n=3) in **c** and **d**. Experiments were repeated two times for **a**.

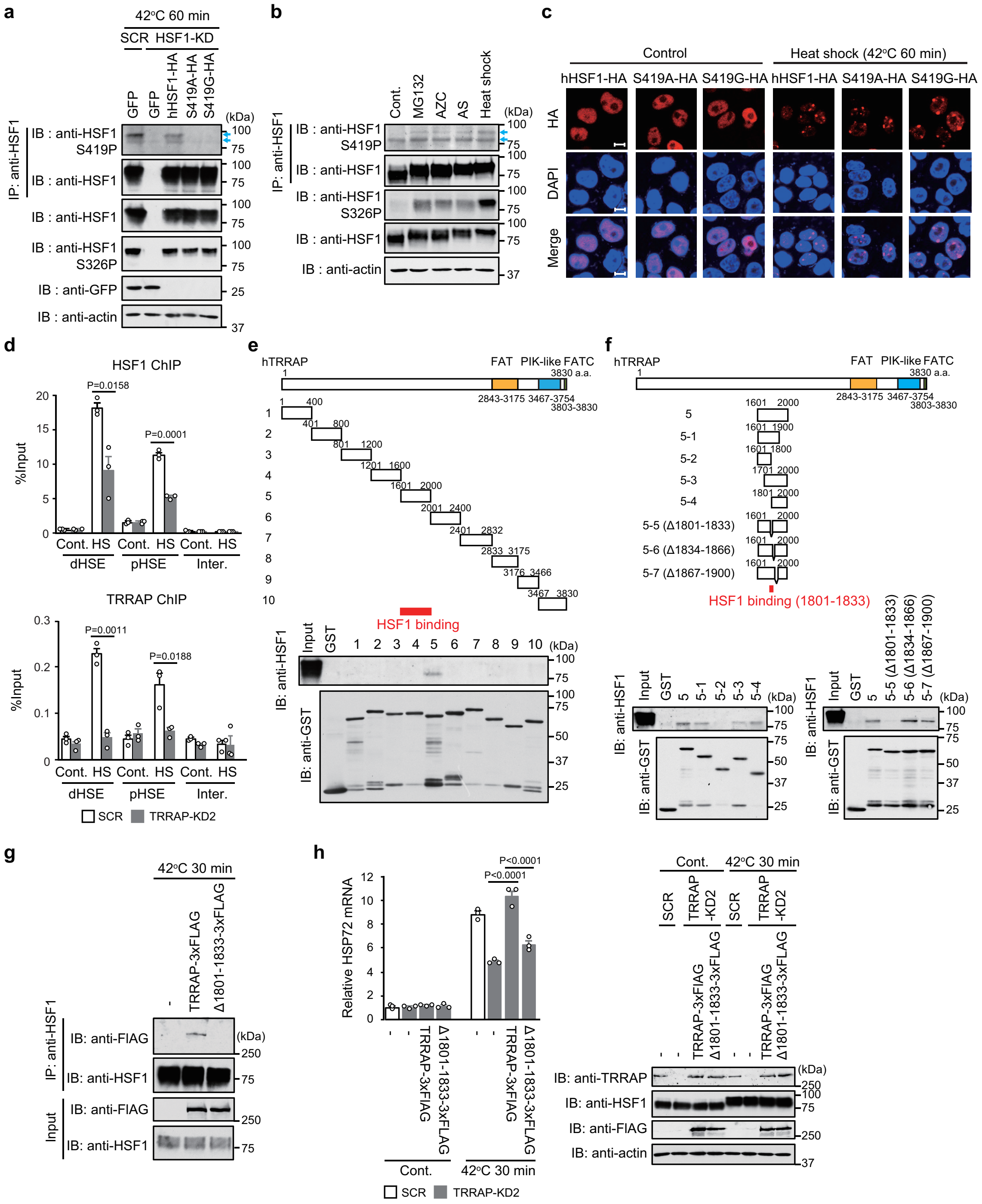
a
42°C 60 min
SCR
HSF1-KD
GFP
GFP
hHSF1-HA
S419A-HA
S419G-HA
(kDa)
IP: anti-HSF1
IB : anti-HSF1 S419P
IB : anti-HSF1
IB : anti-HSF1
IB : anti-HSF1 S326P
IB : anti-GFP
IB : anti-actin
b
Cont.
MG132
AZC
AS
Heat shock
IP: anti-HSF1
IB : anti-HSF1 S419P
IB : anti-HSF1
IB : anti-HSF1 S326P
IB : anti-HSF1
IB : anti-actin
c
Control
Heat shock (42°C 60 min)
hHSF1-HA
S419A-HA
S419G-HA
HA
DAPI
Merge
d
HSF1 ChIP
%Input
P=0.0158
P=0.0001
Cont. HS Cont. HS Cont. HS
dHSE pHSE Inter.
TRRAP ChIP
P=0.0011
P=0.0188
SCR
TRRAP-KD2
e
hTRRAP
FAT
PIK-like
FATC
3830 a.a.
2843-3175
3467-3754
3803-3830
HSF1 binding
IB: anti-HSF1
IB: anti-GST
Input
GST
f
hTRRAP
5-5 (Δ1801-1833)
5-6 (Δ1834-1866)
5-7 (Δ1867-1900)
HSF1 binding (1801-1833)
IB: anti-HSF1
IB: anti-GST
g
42°C 30 min
TRRAP-3xFLAG
Δ1801-1833-3xFLAG
IP: anti-HSF1
IB: anti-FLAG
IB: anti-HSF1
Input
IB: anti-FLAG
IB: anti-HSF1
h
Relative HSP72 mRNA
P<0.0001
P<0.0001
TRRAP-3xFLAG
Δ1801-1833-3xFLAG
Cont.
42°C 30 min
SCR
TRRAP-KD2
Cont.
42°C 30 min
SCR
TRRAP -KD2
IB: anti-TRRAP
IB: anti-HSF1
IB: anti-FLAG
IB: anti-actin

Supplementary Fig. 2. HSF1 is phosphorylated at Ser419 and interacts with TRRAP.

a HeLa cells, in which endogenous HSF1 was replaced with wild-type hHSF1-HA or its mutants, were heat-shocked. Cell extracts were subjected to HSF1 immunoprecipitation and immunoblotting using antibody for HSF1 phospho-S419. Blue arrows indicate the positions of HSF1-S419 phosphorylated bands.

b Cells were treated with 20 μM MG132, 5 mM AZC, or 50 μM As, and cell extracts were subjected to HSF1 immunoprecipitation and immunoblotting using antibody for HSF1 phospho-S419.

c Cells, in which endogenous HSF1 was replaced with wild-type hHSF1-HA or itsu mutants, were heat-shocked. Cells were co-stained with antibody for HA and DAPI, and fluorescence images were merged (Merge). Scale bar, 5 μm.

d HSF1 occupancy in TRRAP KD cells. TRRAP KD cells were heat shock at 42°C for 30 min. ChIP-qPCR of HSF1 and TRRAP on the dHSE, pHSE, and an intergenic region was performed.

e, f Schematic representation of hTRRAP and its fragments fused to GST. TRRAP is a member of the ATM superfamily because it contains the PIK-like (blue box), FAT (orange box), and FATC (green box) domains. The PIK-like, phosphatidylinositol (PI) 3-kinase like domain; FAT, loosely conserved stretches of amino acids in FRAP, ATM and TRRAP; FATC, 35 amino acids of the three FAT factors at the extreme C-terminus[1]. GST pull-down assay was performed using heat-shocked cell extracts, and blotted with HSF1 or GST antibody. The hTRRAP regions that interacted with HSF1 are indicated as red bars.

g Cells transiently overexpressing wild-type hTRRAP-3×FLAG or its deletion mutant were treated with heat shock. Extracts of these cells were subjected to HSF1 immunoprecipitation and immunoblotting.

h TRRAP KD cells transiently overexpressing wild-type hTRRAP-3×FLAG or its deletion mutant were heat-shocked. mRNA levels of HSP72 were quantified by RT-qPCR, and fold induction is shown (left). Cell extracts were subjected to immunoblotting (right).

Norminal *p*-values were determined by two-sided Student's t-test in **d** or one-way ANOVA, followed by Tukey-Kramer test in **h**. Error bars indicate SEM (n=3) in **d** and **h**. Experiments were repeated two times for **a**-**c**, **e**-**g**.

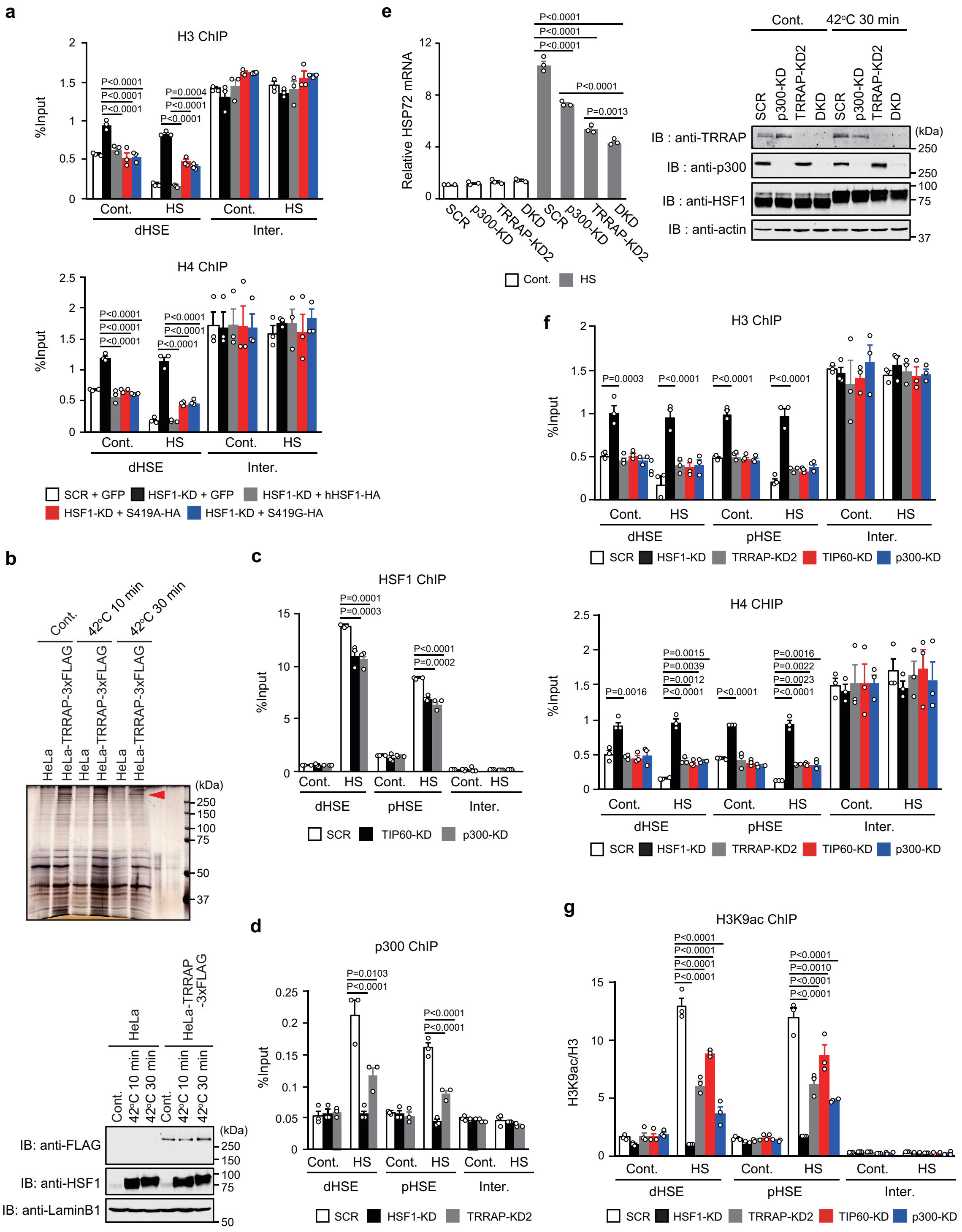
a
H3 ChIP
%Input
P<0.0001
P=0.0004
Cont.
HS
dHSE
Inter.
H4 ChIP
SCR + GFP
HSF1-KD + GFP
HSF1-KD + hHSF1-HA
HSF1-KD + S419A-HA
HSF1-KD + S419G-HA
b
Cont.
42°C 10 min
42°C 30 min
HeLa
HeLa-TRRAP-3xFLAG
(kDa)
IB: anti-FLAG
IB: anti-HSF1
IB: anti-LaminB1
c
HSF1 ChIP
P=0.0001
P=0.0003
P=0.0002
pHSE
SCR
TIP60-KD
p300-KD
d
p300 ChIP
P=0.0103
HSF1-KD
TRRAP-KD2
e
Relative HSP72 mRNA
P=0.0013
SCR
p300-KD
TRRAP-KD2
DKD
Cont.
HS
42°C 30 min
IB : anti-TRRAP
IB : anti-p300
IB : anti-HSF1
IB : anti-actin
f
H3 ChIP
P=0.0003
H4 CHIP
P=0.0016
P=0.0015
P=0.0039
P=0.0012
P=0.0022
P=0.0023
g
H3K9ac ChIP
H3K9ac/H3
P=0.0010

Supplementary Fig. 3. TRRAP-TIP60 complex and p300 are required for histone acetylation at specific residues.

a Occupancy of histone H3 and H4. HeLa cells, in which endogenous HSF1 was replaced with GFP, wild-type hHSF1-HA, or its mutants, were untreated (Cont.) or treated with heat shock at 42°C for 30 min (HS). ChIP-qPCR of H3 and H4 on the dHSE and intergenic region in *HSP72* locus was performed. Occupancy of H3 and H4 was elevated upon HSF1 KD in control cells because HSF1 constitutively binds to *HSP72* promoter and displaces histones from nucleosomes in complex with RPA, the histone chaperone FACT, and the BRG1-containing chromatin remodeling complex[2].

b Identification of TRRAP-interacting proteins. HeLa-hTRRAP-3×FLAG cells (clone 14) were untreated or treated with heat shock, and nuclear extracts were prepared. Proteins co-immunoprecipitated with anti-FLAG antibody were subjected to 8% SDS-PAGE and silver staining or immunoblotting. A red arrowhead indicates the position of hTRRAP-3×FLAG bands.

c TIP60-KD and p300-KD cells were treated with heat shock at 42°C for 30 min. ChIP-qPCR of HSF1 on the dHSE, pHSE, and an intergenic region was performed.

d HSF1-KD and TRRAP-KD cells were treated with heat shock at 42°C for 30 min. ChIP-qPCR of p300 on the dHSE, pHSE, and intergenic region was performed.

e p300-KD, TRRAP-KD and double-KD (DKD) cells were treated with heat shock at 42°C for 30 min. HSP72 mRNA levels were quantified by RT-qPCR, and the levels relative to that in control SCR-treated cells (fold induction) are shown.

f HSF1-KD, TRRAP-KD, TIP60-KD, and p300-KD cells were heat-shocked at 42°C for 30 min. ChIP-qPCR of H3 and H4 on the dHSE, pHSE, and intergenic region was performed.

g HSF1-KD, TRRAP-KD, TIP60-KD, and p300-KD cells were heat-shocked at 42°C for 30 min. ChIP-qPCR of histone H3K9ac was performed.

Norminal *p*-values were determined by one-way ANOVA, followed by Tukey-Kramer test in **a**, **c**-**g**. Error bars indicate SEM (n=3) in **a**, **c**-**g**. Experiments were repeated two times for **b** and **e**.

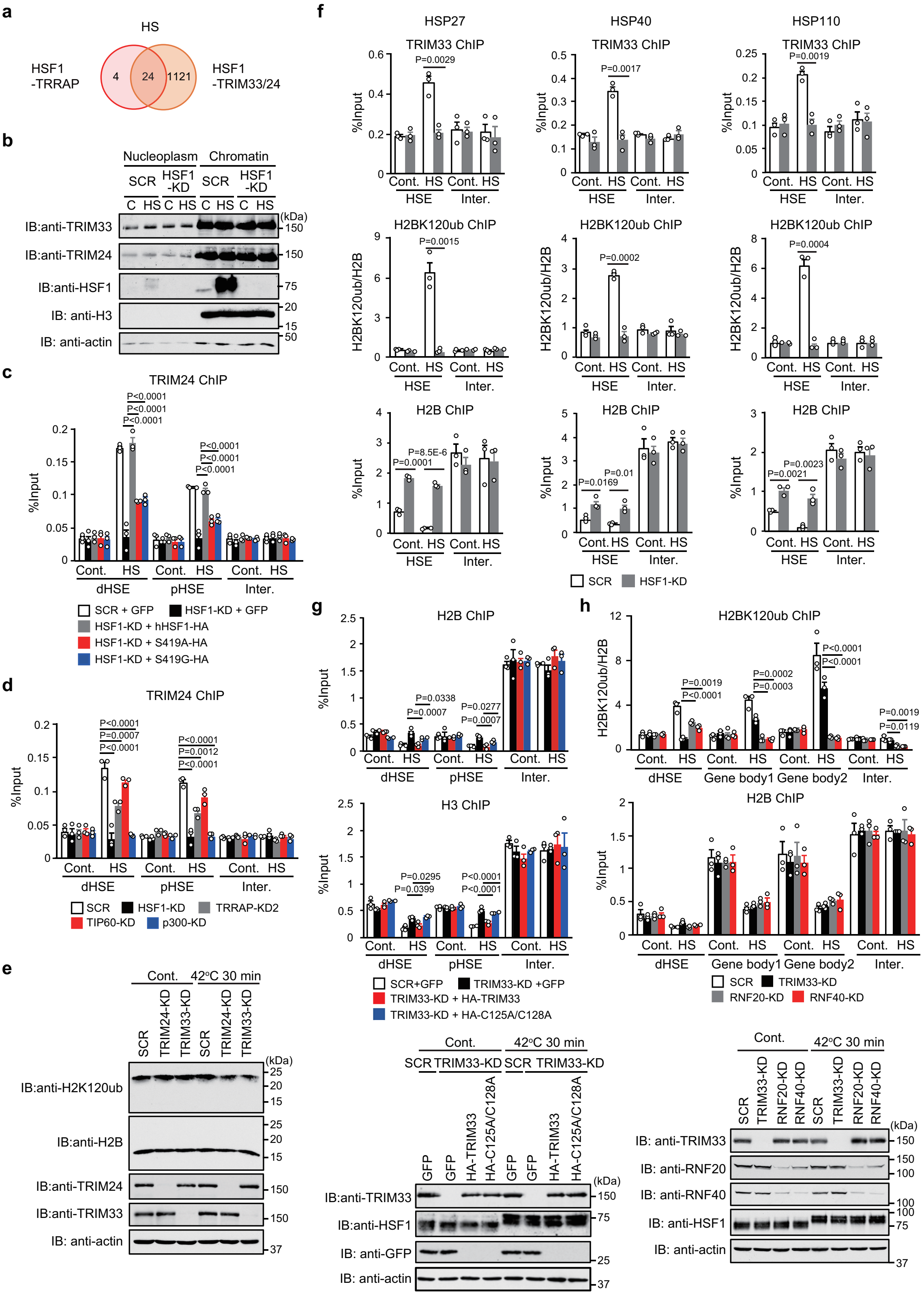
a
HS
HSF1
-TRRAP
4
24
1121
HSF1
-TRIM33/24
b
Nucleoplasm
Chromatin
SCR
HSF1
-KD
C HS C HS C HS C HS
(kDa)
IB:anti-TRIM33
IB:anti-TRIM24
IB:anti-HSF1
IB: anti-H3
IB: anti-actin
c
TRIM24 ChIP
%Input
Cont. HS
dHSE
pHSE
Inter.
SCR + GFP
HSF1-KD + GFP
HSF1-KD + hHSF1-HA
HSF1-KD + S419A-HA
HSF1-KD + S419G-HA
d
TRIM24 ChIP
SCR
HSF1-KD
TRRAP-KD2
TIP60-KD
p300-KD
e
Cont.
42°C 30 min
SCR
TRIM24-KD
TRIM33-KD
IB:anti-H2K120ub
IB:anti-H2B
IB:anti-TRIM24
IB:anti-TRIM33
IB: anti-actin
f
HSP27
HSP40
HSP110
TRIM33 ChIP
H2BK120ub ChIP
H2B ChIP
H2BK120ub/H2B
HSE
Inter.
SCR
HSF1-KD
g
H2B ChIP
H3 ChIP
SCR+GFP
TRIM33-KD +GFP
TRIM33-KD + HA-TRIM33
TRIM33-KD + HA-C125A/C128A
IB:anti-TRIM33
IB:anti-HSF1
IB: anti-GFP
IB: anti-actin
h
H2BK120ub ChIP
H2B ChIP
Gene body1
Gene body2
SCR
TRIM33-KD
RNF20-KD
RNF40-KD
IB: anti-TRIM33
IB: anti-RNF20
IB: anti-RNF40
IB: anti-HSF1
IB: anti-actin

Supplementary Fig. 4. Acetylation-dependent histone H2B mono-ubiquitination by TRIM33 and TRIM24.

a Venn diagram of the HSF1-TRRAP and HSF1-TRIM33-TRIM24 ChIP-seq binding peaks in heat-shocked cells. Numbers of binding peaks are indicated.

b TRIM33 and TRIM24 dominantly bind to chromatin. HSF1-KD HeLa cells were heat-shocked at 42°C for 30 min. Nucleoplasmic (Supernatant) and chromatin (Pellet) fractions were prepared and subjected to immunoblotting. Chromatin associated histone H3 and β-actin are shown as loading controls.

c Cells, in which endogenous HSF1 was replaced with GFP, wild-type hHSF1-HA, or its mutants, were heat-shocked at 42°C for 30 min. ChIP-qPCR of TRIM24 on the dHSE, pHSE, and intergenic region was performed.

d Cells were infected with adenovirus expressing shRNA for HSF1, TRRAP, TIP60, p300, or scrambled RNA (SCR), and were then heat-shocked at 42°C for 30 min. ChIP-qPCR of TRIM24 was performed.

e Cells were infected with adenovirus expressing shRNA for TRIM33 or TRIM24, and were then heat-shocked at 42°C for 30 min. Cell extracts in NP40-lysis buffer was prepared and subjected to immunoblotting.

f Cells were infected with adenovirus expressing shRNA for HSF1 or scrambled RNA (SCR), and were then heat-shocked at 42°C for 30 min. ChIP-qPCR of TRIM33, H2BK120ub, and H2B was performed at the HSEs in the *HSP27*, *HSP40*, and *HSP110* promoters and each intergenic region.

g Cells, in which endogenous TRIM33 was replaced with GFP, wild-type HA-hTRIM33, or the mutant HA-hTRIM33-C125A/C128A were heat-shocked at 42°C for 30 min. ChIP-qPCR of histone H2B and H3 was performed at the HSEs in *HSP72* promoter (upper). Cell extracts were subjected to immunoblotting (lower).

h Cells were infected with adenovirus expressing shRNA for TRIM33, RNF20, or RNF40, and were then heat-shocked at 42°C for 30 min. ChIP-qPCR of TRIM33, H2BK120ub, and H2B was performed at the gene body regions (regions 1 and 2) in *HSP72* locus (upper). Cell extracts were subjected to immunoblotting (lower).

Norminal p-values were determined by one-way ANOVA, followed by Tukey-Kramer test in **c**, **d**, **g**, **h** or two-sided Student's t-test in **f**. Error bars indicate SEM (n=3) in **c**, **d**, and **f**-**h**. Experiments were repeated two times for **b** and **e**.

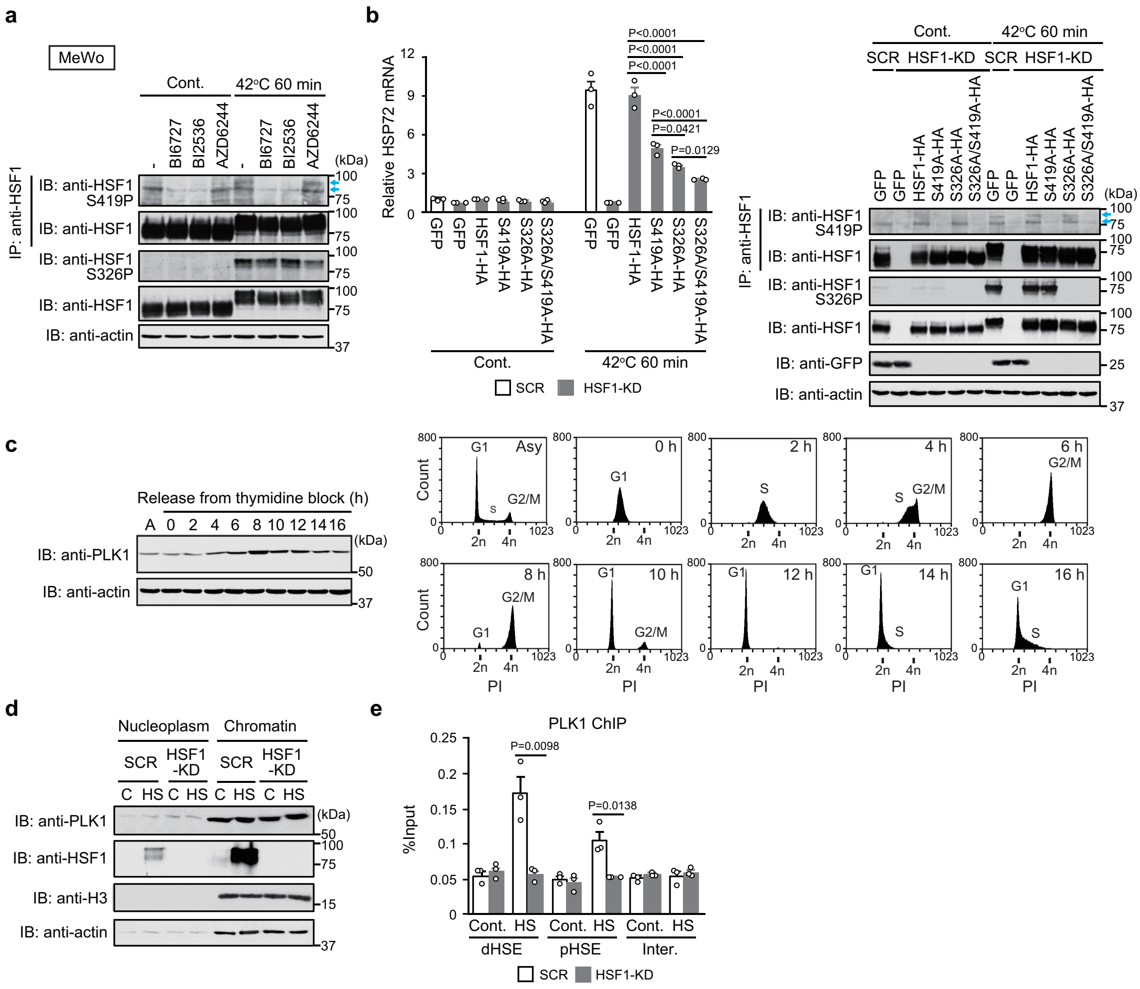
a
MeWo
Cont.
42°C 60 min
-
BI6727
BI2536
AZD6244
(kDa)
IP: anti-HSF1
IB: anti-HSF1 S419P
IB: anti-HSF1
IB: anti-HSF1 S326P
IB: anti-actin
100
75
37
b
Relative HSP72 mRNA
P<0.0001
P=0.0421
P=0.0129
GFP
HSF1-HA
S419A-HA
S326A-HA
S326A/S419A-HA
SCR
HSF1-KD
IB: anti-GFP
25
c
Release from thymidine block (h)
A 0 2 4 6 8 10 12 14 16
IB: anti-PLK1
50
Count
PI
Asy
G1
S
G2/M
2n
4n
1023
0 h
2 h
4 h
6 h
8 h
10 h
12 h
14 h
16 h
d
Nucleoplasm
Chromatin
HSF1 -KD
C HS C HS C HS C HS
IB: anti-H3
15
e
PLK1 ChIP
%Input
P=0.0098
P=0.0138
Cont. HS Cont. HS Cont. HS
dHSE
pHSE
Inter.

Supplementary Fig. 5. PLK1 phosphorylates HSF1-S419 and promotes cell survival during heat shock.

a Human melanoma MeWo cells were pretreated for 3 h with BI6727 (PLK1 inhibitor), BI2536 (PLK inhibitor) or AZD6244 (MEK1/2 inhibitor), and then heat-shocked. Cell extracts were subjected to HSF1 immunoprecipitation and immunoblotting. Blue arrows indicate the positions of HSF1-S419 phosphorylated bands. The intensity of the upper band was markedly enhanced during heat shock.

b HeLa cells, in which endogenous HSF1 was replaced with GFP, wild-type hHSF1-HA, or hHSF1-HA phosphorylation site mutants, were heat-shocked. HSP72 mRNA levels were quantified by RT-qPCR (left). Extracts of these cells were subjected to HSF1 immunoprecipitation and immunoblotting.

c PLK1 protein is expressed at any phase of the cell cycle. Total cellular proteins were prepared by sonication in NP-40 lysis buffer from asynchronous HeLa cells (Asy) and cells that had been doubly thymidine blocked (0 h, G1/S boundary) and released from the double thymidine block for 2 (S phase), 4, 6, 8 (G2/M phase), 12 (G1 phase), 14, and 16 h, and were subjected to immunoblotting using PLK1 or or β-actin antibody (left). Alternatively, these cells were fixed, stained with propidium iodide (PI), and analyzed using a flow cytometer (Cytomics FC500, Beckman Coulter) (right). Peaks of the G1, S, and G2/M phases are indicated.

d PLK1 is dominantly associated with chromatin. HSF1-KD or SCR-treated HeLa cells were heat-shocked at 42°C for 30 min. Nucleoplasmic (Supernatant) and chromatin (Pellet) fractions were prepared and subjected to immunoblotting. Chromatin-associated histone H3 and β-actin are shown as loading controls.

e PLK1 occupancy in *HSP72* promoter during heart shock. Cells were infected for 72 h with adenovirus expressing shRNA for HSF1 (HSF1-KD) or scrambled RNA (SCR), and were then untreated (Cont.) or treated with heat shock at 42°C for 30 min (HS). ChIP-qPCR of PLK1 on the dHSE, pHSE, and intergenic region in *HSP72* locus was performed.

Norminal p-values were determined by one-way ANOVA, followed by Tukey-Kramer test in **b** or two-sided Student's t-test in **e**. Error bars indicate SEM (n=3) in **b** and **e**. Experiments were repeated two times for **a, c** and **d**.

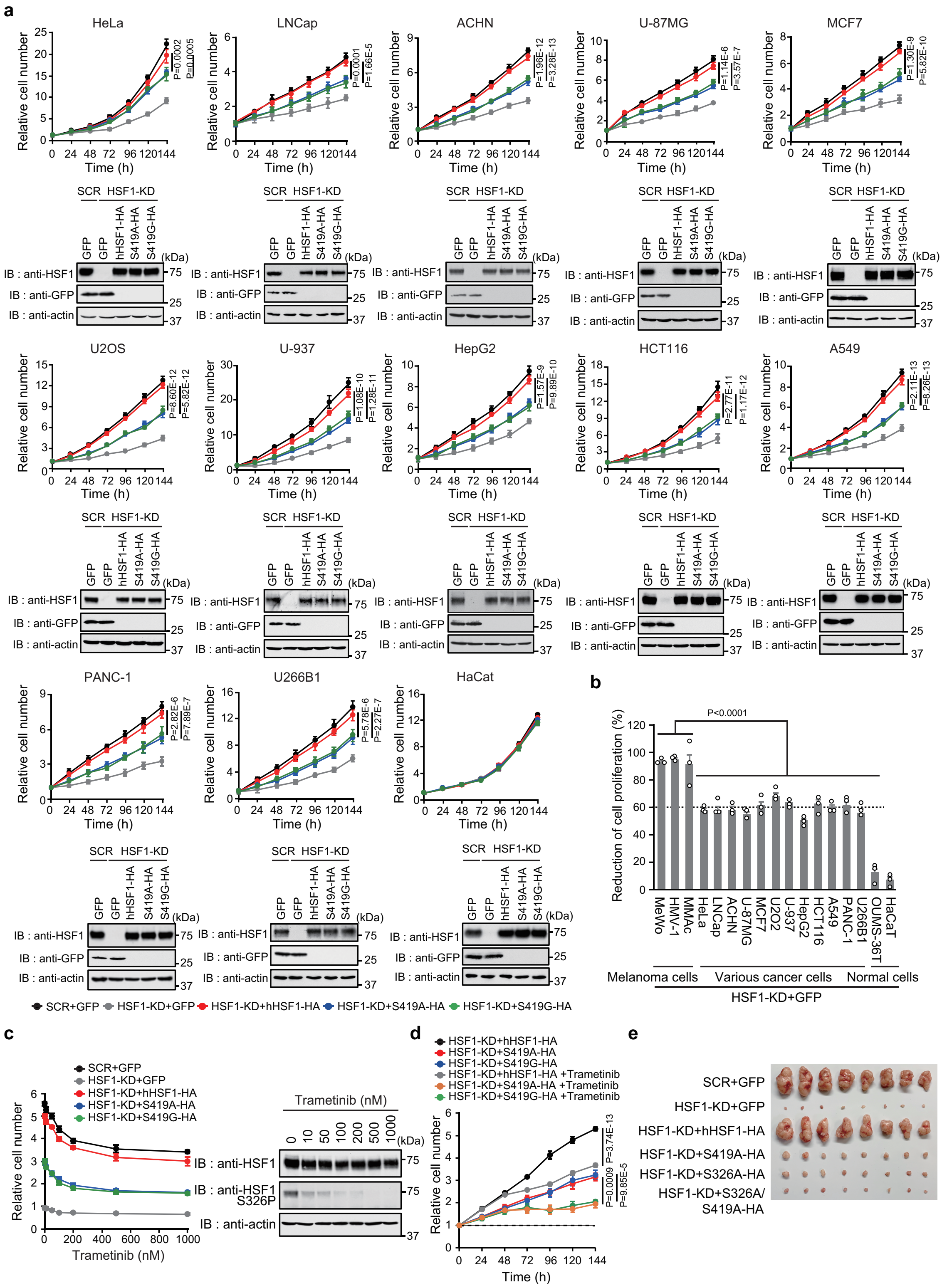
a
HeLa
LNCap
ACHN
U-87MG
MCF7
U2OS
U-937
HepG2
HCT116
A549
PANC-1
U266B1
HaCat
Relative cell number
Time (h)
SCR
HSF1-KD
GFP
hHSF1-HA
S419A-HA
S419G-HA
(kDa)
IB : anti-HSF1
IB : anti-GFP
IB : anti-actin
SCR+GFP
HSF1-KD+GFP
HSF1-KD+hHSF1-HA
HSF1-KD+S419A-HA
HSF1-KD+S419G-HA
b
P<0.0001
Reduction of cell proliferation (%)
MeWo
HMV-1
MMAc
OUMS-36T
Melanoma cells
Various cancer cells
Normal cells
HSF1-KD+GFP
c
Trametinib (nM)
IB : anti-HSF1 S326P
d
HSF1-KD+hHSF1-HA +Trametinib
HSF1-KD+S419A-HA +Trametinib
HSF1-KD+S419G-HA +Trametinib
e
HSF1-KD+S326A-HA
HSF1-KD+S326A/ S419A-HA

Supplementary Fig. 6. Phosphorylation of HSF1-S419 supports the proliferation of various cancer cells.

a Proliferation of various types of cancer cell lines expressing hHSF1-S419A or hHSF1-S419G. Endogenous HSF1 was replaced with GFP, wild-type hHSF1-HA or its mutants in each cancer or normal HaCat cell line. Relative cell numbers at the indicated time points are shown (upper). Extracts of cells were subjected to immunoblotting (lower).

b Comparison of proliferation reduction rates by HSF1 KD in various cancer cells. Cancer cells and normal cells (OUMS-36T-3F and HaCat cells) were treated as described in **a**. Percentages of the reduction in cell proliferation were estimated by comparing the relative cell numbers at 144 h in HSF1 KD cells to that in hHSF1-HA re-expressing cells.

c Concentration-dependent effects of trametinib on the proliferation of MeWo cells expressing wild-type hHSF1 or its S419 mutants. Endogenous HSF1 was replaced with GFP, wild-type hHSF1-HA or its mutants in MeWo cells. These cells were treated for 5 days with the MEK inhibitor trametinib at the indicated concentration. Relative cell numbers are shown (left). Extracts of scrambled RNA-treated, GFP expressing cells were subjected to immunoblotting (right).

d Proliferation profiles of MeWo cells expressing hHSF1-S419 mutants in the presence of trametinib. Endogenous HSF1 was replaced with hHSF1-HA or its mutants in MeWo cells, and maintained in the presence of 200 nM trametinib. Relative cell numbers at the indicated time points are shown. The proliferation of trametinib-treated cells expressing wild-type hHSF1 (48% reduction), hHSF1-S419A (78% reduction), or hHSF1-S419G (76% reduction) was less than that of non-treated cells expressing wild-type hHSF1.

e Tumor formation by melanoma cells expressing hHSF1 phosphorylation site mutants in athymic nude mice. Endogenous HSF1 was replaced with each hHSF1 mutant in MeWo cells. These cells were subcutaneously injected into athymic nude mice, and representative photos of tumors are shown at 22 days after the injection (n=8).

Norminal *p*-values were determined by by two-way ANOVA in **a** and **d**, or one-way ANOVA, followed by Tukey-Kramer test in **b**. Error bars indicate SEM (n=4) in **a**-**d**.

Supplementary Table 1. Primer Sequences Used for Knockdown.

shRNA	Sense strand	Antisense Strand
SCR	5'-GATCCATGTACTGCGCGTGGAGACTTCAAGA GAGTCTCCACGCGCAGTACATTCTTTTTTGGAAA-3'	5'-AGCTTTTCCAAAAAAAAGAGCTATGAAGATTCGATTT CTCTTGAAGTCTCCACGCGCAGTACATG-3'
hHSF1-KD	5'-GATCCAGGTTGTTCATAGTCAGAATTCAAGAGA TTCTGACTATGAACAACCTGCTTTTTTGGAAA-3''	5'-AGCTTTTCCAAAAAAGCAGGTTGTTCATAGTCAGAA TCTCTTGAATTCTGACTATGAACAACCTG-3'
hTRRAP-KD 1	5'-GATCCGCCTGTTCTTTCGCTTTGTACTCAAGAGA TACAAAGCGAAAGAACAGGGCTTTTTTGGAAA -3'	5'-AGCTTTTCCAAAAAAGCCCTGTTCTTTCGCTTTGTA TCTCTTGAATACAAAGCGAAAGAACAGGCG-3'
hTRRAP-KD 2	5'-GATCCGTGTAAGAAAGGGAGAATATTTCAAGAGA ATATTCTCCCTTTCTTACACGTTTTTTGGAAA-3'	5'-AGCTTTTCCAAAAAACGTGTAAGAAAGGGAGAATATT CTCTTGAAATATTCTCCCTTTCTTACACG-3'
hTIP60-KD	5'-GATCCGCAATGAGATTTACCGCAATTCAAGAGA TTGCGGTAAATCTCATTGCCTTTTTTTGGAAA-3'	5'-AGCTTTTCCAAAAAAAGGCAATGAGATTTACCGCAA TCTCTTGAATTGCGGTAAATCTCATTGCG-3'
hp400-KD	5'-GATCCGTGAAGAAGGTTCCCAAGAATTCAAGAGA TTCTTGGGAACCTTCTTCATTTTTTGGAAA-3'	5'-AGCTTTTCCAAAAAATGAAGAAGGTTCCCAAGAA TCTCTTGAATTCTTGGGAACCTTCTTCAG-3'
hp300-KD	5'-GATCCGCAGCTCAACCATCCACTATTCAAGAGA TAGTGGATGGTTGAGCTGCTGTTTTTTGGAAA-3'	5'-AGCTTTTCCAAAAAACAGCAGCTCAACCATCCACTA TCTCTTGAATAGTGGATGGTTGAGCTGCG-3'
hCBP-KD	5'-GATCCGCAGCAGCCAGCATTGATATTCAAGAGA TATCAATGCTGGCTGCTGCCTTTTTTTGGAAA-3'	5'-AGCTTTTCCAAAAAAAGGCAGCAGCCAGCATTGATA TCTCTTGAATATCAAGCTGGCTGCTGCG-3'
hGCN5-KD	5'-GATCCGAAGCTGATTGAGCGCAAATTCAAGAGA TTTGCGCTCAATCAGCTTCTTTTTTGGAAA-3'	5'-AGCTTTTCCAAAAAAGAAGCTGATTGAGCGCAAA TCTCTTGAATTTGCGCTCAATCAGCTTCG-3'
hPCAF-KD	5'-GATCCGTCGCCGTGAAGAAAGCGCATTCAAGAG ATGCGCTTTCTTCACGGCGATTTTTTGGAAA-3'	5'-AGCTTTTCCAAAAAATCGCCGTGAAGAAAGCGCA TCTCTTGAATGCGCTTTCTTCACGGCGACG-3'
hTRIM24-KD	5'-GATCCGCAGGTGGAACAGGATATTCTCAAGAGA AATATCCTGTTCCACCTGCTTTTTTGGAAA-3'	5'-AGCTTTTCCAAAAAAGCAGGTGGAACAGGATATT TCTCTTGAGAATATCCTGTTCCACCTGCG-3'
hTRIM33-KD	5'-GATCCAAGCGACTGATTACTTTCCTTCAAGAGA GGAAAGTAATCAGTCGCTTGCTTTTTTGGAAA-3'	5'-AGCTTTTCCAAAAAAGCAAGCGACTGATTACTTTCC TCTCTTGAAGGAAAGTAATCAGTCGCTTG-3'
hPLK1-KD	5'-GATCCGATCACCCTCCTTAAATATCTCAAGAGA ATATTTAAGGAGGGTGATCTTTTTTTGGAAA-3'	5'-AGCTTTTCCAAAAAAAGATCACCCTCCTTAAATAT TCTCTTGAGATATTTAAGGAGGGTGATCG-3'
hCSNK1A1-KD	5'-GATCCATCTATTTGGCGATCAACACTCAAGAGA TGTTGATCGCCAAATAGATGTTTTTTTGGAAA-3'	5'-AGCTTTTCCAAAAAAACATCTATTTGGCGATCAACA TCTCTTGAGTGTTGATCGCCAAATAGATG-3'
hNEK7-KD	5'-GATCCGCCGACAGTTAGTTAATATGCTCAAGAGA CATATTAACTAACTGTCGGAGTTTTTTGGAAA-3'	5'-AGCTTTTCCAAAAAACTCCGACAGTTAGTTAATATG TCTCTTGAGCATATTAACTAACTGTCGGCG-3'
hRNF20-KD	5'-GATCCAGGCAGCTGTTGAAGATTCCTCAAGAGA GAATCTTCAACAGCTGCCTTCTTTTTTGGAAA-3'	5'-AGCTTTTCCAAAAAAGAAGGCAGCTGTTGAAGATTC TCTCTTGAGGAATCTTCAACAGCTGCCTG-3'
hRNF40-KD	5'-GATCCAGCTTAACTCTGGCTACTACTCAAGAGA TAGTAGCCAGAGTTAAGCTGCTTTTTTGGAAA-3'	5'-AGCTTTTCCAAAAAAGCAGCTTAACTCTGGCTACTA TCTCTTGAGTAGTAGCCAGAGTTAAGCTG-3'

Supplementary Table 2. Primer Sequences Used for ChIP-qPCR.

CHIP-qPCR	Forward primer	Reverse Primer
HSP72 dHSE	5'-CCCTGTCCCCTCCAGTGAAT-3'	5'-TCCTTGGACCAATCAGAGG-3'
HSP72 pHSE	5'-GGCAGGACGGGAGGCGAAAC-3'	5'-CTTTTCCCTTCTGAGCCAATCA-3'
HSP72 Pausing	5'-GCTGCGACAGTCCACTACCTT-3'	5'-CTGGGAAGCCTTGGGACAA-3'
HSP72 Gene body 1	5'-GGGTGGGGAGGACTTTGACAACAGG-3'	5'-TGGCTGATGTCCTTCTTGTG-3'
HSP72 Gene body 2	5'-GACGCAGATCTTCACCACCT-3'	5'-GCCCCAACAGATTGTTGTCT-3'
HSP72 Inter.	5'-GGAATGGAAGGAGTGTCCTAAA-3'	5'-TAACTCAGCAAGCCAGTGAAT-3'
HSP27 HSE	5'-GCTTTCCTTAACGAGAGAAG-3'	5'-GCACTCCTCCCCCAGCGTTC-3'
HSP27 Inter.	5'-GTCCAGAGACCCACATCTGG-3'	5'-GGCTCACATCATCTGAGCTG-3'
HSP40 HSE	5'-GCGGAAGGTTCTGGAGGGG-3'	5'-GGACTCTATATACCCGTCCG-3'
HSP40 Inter.	5'-GTCACAAACATAAAGGTGCA-3'	5'-GTAGGTTGCTTGGCCGTGTT-3'
HSP110 HSE	5'-GAACTTTCCAGAATCTGCGG-3'	5'-GAAGGCTCGAGAAGAAGGAA-3'
HSP110 Inter.	5'-GTGAATCCCCATTTCCTCAT-3'	5'-GCACTAGAGGAATCCACCAT-3'

Supplementary Table 3. Primer Sequences Used for RT-qPCR.

Real-time PCR	Forward primer	Reverse primer	Probe
hHSP72	5'-GCCGAGAAGGACGAGTTTGA-3'	5'-CCTGGTACAGTCCGCTGATGA-3'	5'-FAM-TTACACACCTGCTCCAGCTC CTTCCTCTT-TAMRA-3'
hGAPDH	5'-GTTCGACAGTCAGCCGCATC-3'	5'-GGAATTTGCCATGGGTGGA-3'	5'-FAM-ACCAGGCGCCCAATACGAC CAA-TAMRA-3'

Supplementary Table 4. Primer Sequences Used for RT-qPCR.

RT-PCR	Forward primer	Reverse primer
hHSP110	5'-AAAAAATAGAACAATCGGAG-3'	5'-TGAAGTTAGACACCGTATTGTTTGC-3'
hHSP90α	5'-TGGACAGCAAACATGGAGAG-3'	5'-AGACAGGAGCGCAGTTTCAT-3'
hHSP40	5'-ACCCATTCTCTGGCTTCCCTAT-3'	5'-CGGGAGCGGCCAAAG-3'
hHSP27	5’-CTGACGGTCAAGACCAAGGATG-3’	5’-GTGTATTTCCGCGTGAAGCACC-3’
hactin	5'-TGGATCAGCAAGCAGGAGTATG-3'	5'-GCATTGCGGTGGACGAT-3'

Supplementary References

1. Murr, R., Vaissière, T., Sawan, C., Shukla, V. & Herceg, Z. Orchestration of chromatin-based processes: mind the TRRAP. *Oncogene* **26,** 5358-5372 (2007).
2. Fujimoto, M., Takaki, E., Takii, R., Tan, K., Prakasam, R., Hayashida, N., Iemura, S., Natsume, T. & Nakai, A. RPA assists HSF1 access to nucleosomal DNA by recruiting histone chaperone FACT. *Mol. Cell* **48,** 182-194 (2012).
