# Supplementary material for: HSF1 phosphorylation establishes an active chromatin state via the TRRAP–TIP60 complex and promotes tumorigenesis
Source: Nat Commun. 2022 Jul 29;13:4355. doi: 10.1038/s41467-022-32034-4 (PMC9338313; doi:10.1038/s41467-022-32034-4)
Supplement: Supplementary file 3 — Description of Additional Supplementary [file 41467_2022_32034_MOESM3_ESM.pdf]

Description of Additional Supplementary Files

Supplementary Data 1. HSF1-interacting proteins enriched upon heat shock.

Supplementary Data 2. Time-dependent abundance profile of HSF1-interacting proteins during heat shock.

Supplementary Data 3. TRRAP-interacting proteins during heat shock.
