# Supplementary material for: HSF1 phosphorylation establishes an active chromatin state via the TRRAP–TIP60 complex and promotes tumorigenesis
Source: Nat Commun. 2022 Jul 29;13:4355. doi: 10.1038/s41467-022-32034-4 (PMC9338313; doi:10.1038/s41467-022-32034-4)
Supplement: Supplementary file 7 — Reporting Summary [file 41467_2022_32034_MOESM7_ESM.pdf]

nature portfolio

Corresponding author(s): Akira Nakai

Last updated by author(s): Jul 7, 2022

Reporting Summary

Nature Portfolio wishes to improve the reproducibility of the work that we publish. This form provides structure for consistency and transparency in reporting. For further information on Nature Portfolio policies, see our Editorial Policies and the Editorial Policy Checklist.

Statistics

For all statistical analyses, confirm that the following items are present in the figure legend, table legend, main text, or Methods section.

n/a	Confirmed	
☐	☒	The exact sample size (*n*) for each experimental group/condition, given as a discrete number and unit of measurement
☐	☒	A statement on whether measurements were taken from distinct samples or whether the same sample was measured repeatedly
☐	☒	The statistical test(s) used AND whether they are one- or two-sided<br>*Only common tests should be described solely by name; describe more complex techniques in the Methods section.*
☒	☐	A description of all covariates tested
☒	☐	A description of any assumptions or corrections, such as tests of normality and adjustment for multiple comparisons
☐	☒	A full description of the statistical parameters including central tendency (e.g. means) or other basic estimates (e.g. regression coefficient) AND variation (e.g. standard deviation) or associated estimates of uncertainty (e.g. confidence intervals)
☐	☒	For null hypothesis testing, the test statistic (e.g. F, t, r) with confidence intervals, effect sizes, degrees of freedom and P value noted<br>*Give P values as exact values whenever suitable.*
☒	☐	For Bayesian analysis, information on the choice of priors and Markov chain Monte Carlo settings
☒	☐	For hierarchical and complex designs, identification of the appropriate level for tests and full reporting of outcomes
☒	☐	Estimates of effect sizes (e.g. Cohen's d, Pearson's r), indicating how they were calculated

Our web collection on statistics for biologists contains articles on many of the points above.

Software and code

Policy information about availability of computer code

Data collection	No software was used.
Data analysis	ImageJ version 1.53k ; https://imagej.nih.gov/ij/ (Schneider et al., 2012).<br>Mascot version 2.8.0.1 ; https://www.matrixscience.com/server.html (MatrixScience).<br>MaxQuant version 2.0.3.1; https://www.maxquant.org (Max planck institute of biochemistry).

For manuscripts utilizing custom algorithms or software that are central to the research but not yet described in published literature, software must be made available to editors and reviewers. We strongly encourage code deposition in a community repository (e.g. GitHub). See the Nature Portfolio guidelines for submitting code & software for further information.

Data

Policy information about availability of data

All manuscripts must include a data availability statement. This statement should provide the following information, where applicable:

- Accession codes, unique identifiers, or web links for publicly available datasets
- A description of any restrictions on data availability
- For clinical datasets or third party data, please ensure that the statement adheres to our policy

The ChIP-MS data and TRRAP-interacting proteins MS data generated during this study are available at jPOSTrepo (Japan ProteOme STandard Repository) with PXD accession code: PXD031821 (http://proteomecentral.proteomexchange.org/cgi/GetDataset?ID=PXD031821) and PXD accession code: PXD031822 (http://proteomecentral.proteomexchange.org/cgi/GetDataset?ID=PXD031822), respectively. The ChIP-seq data are available at NCBI BioProject with BioProject ID: PRJNA811704 (https://www.ncbi.nlm.nih.gov/bioproject/PRJNA811704). Any additional information required to reanalyze the data reported in this paper is available from the lead contact upon request.

Field-specific reporting

Please select the one below that is the best fit for your research. If you are not sure, read the appropriate sections before making your selection.

☒ Life sciences ☐ Behavioural & social sciences ☐ Ecological, evolutionary & environmental sciences

For a reference copy of the document with all sections, see nature.com/documents/nr-reporting-summary-flat.pdf

Life sciences study design

All studies must disclose on these points even when the disclosure is negative.

Sample size	All experiments were performed at least three times and significance was determined using Student's t-test, two-way ANOVA, or one-way ANOVA, followed by Tukey-Kramer post hoc test.
Data exclusions	No data were excluded form the analyses.
Replication	All experiments were performed successfully at least twice, and representative data are shown (e.g. gel images).
Randomization	This is not relevant to our study. All of the experiments were conducted using cell lines. We also inoculated differently treated MeWo melanoma cells into male athymic nude mice with the same genetic background.
Blinding	Blinding was not relevant to our study. The phenotypes were quite obvious that observer can be sure without blind test.

Reporting for specific materials, systems and methods

We require information from authors about some types of materials, experimental systems and methods used in many studies. Here, indicate whether each material, system or method listed is relevant to your study. If you are not sure if a list item applies to your research, read the appropriate section before selecting a response.

Materials & experimental systems

n/a	Involved in the study	
☐	☒	Antibodies
☐	☒	Eukaryotic cell lines
☒	☐	Palaeontology and archaeology
☐	☒	Animals and other organisms
☒	☐	Human research participants
☒	☐	Clinical data
☒	☐	Dual use research of concern

Methods

n/a	Involved in the study	
☐	☒	ChIP-seq
☒	☐	Flow cytometry
☒	☐	MRI-based neuroimaging

Antibodies

Antibodies used	Antibody used for western blot: rabbit polyclonal anti-mHSF1n (1/1000, Akira Nakai Lab Yamaguchi University School of Medicine), rabbit polyclonal anti-hTRRAP-2 (1/1000, Akira Nakai Lab Yamaguchi University School of Medicine), rabbit polyclonal anti-hTRIM24-3 (1/1000, Akira Nakai Lab Yamaguchi University School of Medicine), rabbit polyclonal anti-hTRIM33-2 (1/1000, Akira Nakai Lab Yamaguchi University School of Medicine), rabbit polyclonal anti-hPLK1-1 (1/1000, Akira Nakai Lab Yamaguchi University School of Medicine), rabbit polyclonal anti-GST (1/1000, Akira Nakai Lab Yamaguchi University School of Medicine), rabbit polyclonal anti-mHSP110a (1/1000, Akira Nakai Lab Yamaguchi University School of Medicine), rabbit polyclonal anti-hHSP90d (1/1000, Akira Nakai Lab Yamaguchi University School of Medicine), anti-hHSP40a (1/1000, Akira Nakai Lab Yamaguchi University School of Medicine), rabbit polyclonal anti-p300 (1/1000, Santa Cruz Biotechnology, SC-585), rabbit polyclonal anti-p400 (1/1000, Novus, NB200-210), rabbit polyclonal anti-Histone H2B (1/1000, Abcam, ab1790), rabbit monoclonal anti-Ubiquityl-Histone H2B (Lys120) (D11) XP (1/1000, Cell Signaling Technology, 5546), rabbit polyclonal anti-RNF40 (1/1000, GeneTex, GTX115295, N1 N2), rabbit polyclonal anti-Histone H3 (1/1000, Abcam, ab1791), rabbit polyclonal anti-Histone H3 (acetyl K18) (1/1000, Abcam, ab1191), rabbit polyclonal anti-Lamin B1 (1/1000, Abcam, Ab16048), rabbit polyclonal anti-HSF1 (phospho S326) (1/1000, Abcam, ab115702), rabbit polyclonal anti-HSF1 phospho-S419b (1/1000, Akira Nakai Lab Yamaguchi University School of Medicine, made by Cosmo Bio Co., Otaru, Hokkaido, Japan), mouse monoclonal anti-hPLK1 (1/1000, Santa Cruz Biotechnology, sc-17783, F-8), mouse monoclonal anti-HSP70/HSC70 (1/1000, Santa Cruz Biotechnology, sc-24, W27), mouse monoclonal anti-Ub (1/1000, Santa Cruz Biotechnology, sc-8017, AC P4D1), mouse monoclonal anti-casein kinase l alpha (1/1000, Santa Cruz Biotechnology, sc-74582, H-7), mouse monoclonal anti-NEK7 (1/1000, Santa Cruz Biotechnology, sc-393539, B-5), mouse monoclonal anti-RNF20 (1/1000, Santa Cruz Biotechnology, sc-517358), mouse monoclonal anti-β-actin (1/1000, Milipore sigma, A5441), mouse monoclonal anti-GFP (1/1000, Nacalai Tesque, GF200), rat monoclonal anti-HA high affinity (1/1000, Roche, ROAHAHA), mouse monoclonal anti-FLAG (1/1000, Sigma-Aldrich, F3165, clone M2), rat monoclonal anti-HA high affinity (1/1000, Roche, ROAHAHA), goat polyclonal anti-TIP60 (1/200, Santa Cruz Biotechnology, sc-5725, N-17), HRP-conjugated goat anti-rabbit IgG (whole molecule) secondary antibody (1/2000, MP Biomedicals, 55689), HRP-conjugated goat anti-mouse IgG (H+L) (1/1000, Jackson, 115-035-003), HRP-conjugated rabbit anti-goat IgG (whole molecule) secondary antibody (1/1000, MP Biomedicals, 55363), HRP-conjugated goat anti-rat IgG (H+L) (1/1000, Jackson, 112-035-003).

Antibody used for ChIP: anti-HSF1 (Millipore Sigma, ABE1044, 2 µl), anti-mHSF1j (Akira Nakai Lab Yamaguchi University School of Medicine, 2 µl), anti-RNA polymerase II (Millipore sigma, 05-623, 2 µl), anti-hTRRAP-2 (Akira Nakai Lab Yamaguchi University School of Medicine, 2 µl), anti-Histone H3 (Abcam, ab1791, 2 µl), anti-acetyl-Histone H3 (Millipore Sigma, 06-599, 2 µl), anti-Histone H4 (Abcam, ab7311, 2 µl), anti-Histone H4ac (pan-acetyl) (Active Motif, 39925, 2 µl), anti-TIP60 (Santa Cruz Biotechnology, sc-5725, N-17, 10 µl), anti-p300 (Santa Cruz Biotechnology, SC-585, 10 µl), anti-Histone H3 (acetyl Lys9) (Millipore Sigma, 07-352, 2 µl), anti-Histone H3 (acetyl K18) (Abcam, ab1191, 2 µl), anti-Histone H3 (acetyl K23) (Abcam, ab177275, 2 µl), anti-H3K27ac (Abcam, ab4729, 2 µl), anti-Histone H4 (acetyl K16) (Abcam, ab109463, 2 µl), anti-hTRIM24-3 (Akira Nakai Lab Yamaguchi University School of Medicine, 2 µl), anti-hTRIM33-2 (Akira Nakai Lab Yamaguchi University School of Medicine, 2 µl), anti-Histone H2B (Abcam, ab1790, 2 µl), anti-Ubiquityl-Histone H2B (Lys120) (D11) XP (Cell Signaling Technology, 5546, 4 µl), anti-Histone H3 (tri methyl K4) (Abcam, ab213224, 2 µl), anti-hPLK1-1 (Akira Nakai Lab Yamaguchi University School of Medicine, 2 µl).

Antibody used for Immunofluorescence: rat monoclonal anti-HA high affinity (1/1000, Roche, ROAHAHA), goat anti-rat IgG (H+L) cross-adsorbed secondary antibody, Alexa FluorTM546 (1/1000, Thermo Fisher Scientific, A-11081).

Validation

The antibodies were generated by Akira Nakai Lab, Yamaguchi University School of Medicine.
anti-mHSF1j, anti-mHSF1n, anti-hTRRAP-2, hTRIM24-3, hTRIM33-2, anti-hPLK1-1, anti-GST, anti-mHSP110a, anti-hHSP90d, anti-hHSP40a.

The antibodies were generated by Cosmo Bio Co., Otaru, Hokkaido, Japan.
anti-HSF1 phospho-S419b

The antibodies were purchased from commercial sources.
anti-p300 (Santa Cruz Biotechnology, SC-585), https://datasheets.scbt.com/sc-585.pdf
anti-p400 (Novus, NB200-210), https://www.novusbio.com/products/p400-antibody_nb200-210
anti-Ubiquityl-Histone H2B (Lys120) (D11) XP (Cell Signaling Technology, 5546), https://www.cellsignal.jp/products/primary-antibodies/ubiquityl-histone-h2b-lys120-d11-xp-rabbit-mab/5546
anti-RNF40 (GeneTex, GTX115295, N1 N2), https://www.genetex.com/Product/Detail/RNF40-antibody-N1N2-N-term/GTX115295
anti-Histone H3 (Abcam, ab1791), https://www.abcam.com/histone-h3-antibody-nuclear-marker-and-chip-grade-ab1791.html
anti-Histone H3 (acetyl K18) (Abcam, ab1191), https://www.abcam.com/histone-h3-acetyl-k18-antibody-chip-grade-ab1191.html
anti-HSF1 (phospho S326) (Abcam, ab115702), https://www.abcam.com/hsf1-phospho-s326-antibody-ab115702.html
anti-hPLK1 (Santa Cruz Biotechnology, sc-17783, F-8), https://datasheets.scbt.com/sc-17783.pdf
anti-HSP70/HSC70 (1:1000, Santa Cruz Biotechnology, sc-24, W27), https://datasheets.scbt.com/sc-24.pdf
anti-Ub (Santa Cruz Biotechnology, sc-8017, AC P4D1), https://datasheets.scbt.com/sc-8017.pdf
anti-casein kinase l alpha (Santa Cruz Biotechnology, sc-74582, H-7), https://datasheets.scbt.com/sc-74582.pdf
anti-NEK7 (Santa Cruz Biotechnology, sc-393539, B-5), https://datasheets.scbt.com/sc-393539.pdf
anti-RNF20 (Santa Cruz Biotechnology, sc-517358), https://datasheets.scbt.com/sc-517358.pdf
anti-β-actin (Milipore Sigma, A5441), https://www.sigmaaldrich.com/US/en/product/sigma/a5441
anti-GFP (Nacalai Tesque, GF200), https://www.nacalai.co.jp/ss/ec2/ec-srchdetl.cfm?HP=1&l=JP&lc=1&syohin=0436366&syubetsu=3&catalog=&SiireC=&MakerC=&yoro=&mv=1
anti-HA high affinity (Roche, ROAHAHA), https://www.sigmaaldrich.com/US/en/product/roche/roahaha?gclid=Cj0KCQjwzLCVBhD3ARIsAPKYTcQ0aNnutkNZYhuetrj1vATWyWGZ9TUxLyrRSusVzIpcnpffpgPS-ykaAqT5EALw_wcB
anti-TIP60 (Santa Cruz Biotechnology, sc-5725, N-17), https://datasheets.scbt.com/sc-5725.pdf
anti-HSF1 (Millipore Sigma, ABE1044), https://www.emdmillipore.com/US/en/product/Anti-HSF-1-Antibody,MM_NF-ABE1044?bd=1
anti-RNA polymerase II (Millipore Sigma 05-623), https://www.emdmillipore.com/US/en/product/Anti-RNA-polymerase-II-Antibody-clone-CTD4H8,MM_NF-05-623
anti-acetyl-Histone H3 (Millipore Sigma 06-599), https://www.emdmillipore.com/US/en/product/Anti-acetyl-Histone-H3-Antibody,MM_NF-06-599
anti-Histone H4 (Abcam ab7311), https://www.abcam.com/histone-h4-antibody-chip-grade-ab7311.html
anti-Histone H4ac (pan-acetyl) (Active Motif 39925), https://www.activemotif.com/catalog/details/39925
anti-Histone H3 (acetyl Lys9) (Millipore Sigma 07-352), https://www.emdmillipore.com/US/en/product/Anti-acetyl-Histone-H3-Lys9-Antibody,MM_NF-07-352
anti-Histone H3 (acetyl K23) (Abcam, ab177275), https://www.abcam.com/histone-h3-acetyl-k23-antibody-epr17712-chip-grade-ab177275.html
anti-H3K27ac (Abcam, ab4729), https://www.abcam.com/histone-h3-acetyl-k27-antibody-chip-grade-ab4729.html
anti-Histone H4 (acetyl K16) (Abcam, ab109463), https://www.abcam.com/histone-h4-acetyl-k16-antibody-epr1004-ab109463.html
anti-Ubiquityl-Histone H2B (Lys120) (D11) XP (Cell Signaling Technology, 5546), https://www.cellsignal.co.uk/products/primary-antibodies/ubiquityl-histone-h2b-lys120-d11-xp-rabbit-mab/5546?N=0+102236+4294956287&Nrpp=200&No=2600&fromPage=plp
anti-Histone H3 (tri methyl K4) (Abcam, ab213224), https://www.abcam.com/histone-h3-tri-methyl-k4-antibody-epr20551-225-chip-grade-ab213224.html
anti-FLAG (Sigma-Aldrich, F3165, clone M2), https://www.sigmaaldrich.com/JP/en/product/sigma/f3165
HRP-conjugated goat anti-Rabbit IgG (Whole Molecule) Secondary Antibody (MP Biomedicals, 55689), https://www.mpbio.com/jp/0855689-peroxidase-conjugated-goat-affinity-purified-antibody-to-rabbit-igg-whole-molecule
HRP-conjugated goat anti-Mouse IgG (H+L) (Jackson, 115-035-003), https://www.jacksonimmuno.com/catalog/products/115-035-003
HRP-conjugated rabbit anti-Goat IgG (Whole Molecule) Secondary Antibody (MP Biomedicals, 55363), https://www.mpbio.com/jp/0855363-peroxidase-conjugated-rabbit-affinity-purified-antibody-to-goat-igg-whole-molecule
HRP-conjugated goat anti Rat IgG (H+L) (Jackson, 112-035-003), https://www.jacksonimmuno.com/catalog/products/112-035-003
goat anti-Rat IgG (H+L) Cross-Adsorbed Secondary Antibody, Alexa FluorTM546 (Thermo Fisher Scientific, A-11081), https://www.thermofisher.com/antibody/product/Goat-anti-Rat-IgG-H-L-Cross-Adsorbed-Secondary-Antibody-Polyclonal/A-11081

All antibodies were validated by the manufacturer or our laboratory. Antibody specificity was evaluated using the proper negative control.

Eukaryotic cell lines

Policy information about cell lines

Cell line source(s)	Human: HeLa cells ATCC CRM-CCL-2<br>Human: HEK293 cells ATCC CRL-1573<br>Human: MeWo cells ATCC HTB-65<br>Human: MMAc cells RIKEN BRC RCB0808<br>Human: OUMS-36T-3F JCRB Cell Bank JCRB1006.3F<br>Human: HMV-1 cells Cell Resource Center for Biomedical Research, Tohoku University TKG0302<br>Human: LNCap cells RIKEN BRC RCB2144<br>Human: ACHN cells ATCC CRL-1611<br>Human: U-87MG cells ATCC HTB-14<br>Human: MCF7 cells RIKEN BRC RCB1904<br>Human: U2OS cells ATCC HTB-96<br>Human: U-937 cells ATCC CRL-1593.2<br>Human: HepG2 cells RIKEN BRC RCB1648<br>Human: HCT116 cells RIKEN BRC RCB2979<br>Human: A549 cells RIKEN BRC RCB0098<br>Human: PANC-1 cells RIKEN BRC RCB2095<br>Human: U266B1 cells ATCC TIB-196<br>Human: HaCat cells Cell Lines Service Cat#300493-SF
Authentication	All cell lines were routinely authenticated by morphological examination using microscopy.
Mycoplasma contamination	The cell lines were not tested for mycoplasma contamination.
Commonly misidentified lines<br>(See ICLAC register)	No cell line used is listed in the database of commonly misidentified cell lines.

Animals and other organisms

Policy information about studies involving animals; ARRIVE guidelines recommended for reporting animal research

Laboratory animals	5-week-old male athymic nude mice (BALB/cSlc-nu/nu, SLC Inc., Japan).
Wild animals	This study did not involve wild animals.
Field-collected samples	This study did not involve samples collected from the field.
Ethics oversight	All experimental protocols related to these mice were approved by the Committee for Ethics on Animal Experiments of Yamaguchi University Graduate School of Medicine.

Note that full information on the approval of the study protocol must also be provided in the manuscript.

ChIP-seq

Data deposition

☒ Confirm that both raw and final processed data have been deposited in a public database such as GEO.

☒ Confirm that you have deposited or provided access to graph files (e.g. BED files) for the called peaks.

Data access links<br>*May remain private before publication.*	The ChIP-seq data are available at NCBI BioProject with BioProject ID: PRJNA811704 (https://www.ncbi.nlm.nih.gov/bioproject/PRJNA811704).
Files in database submission	Supplementary Data 1 and 2.
Genome browser session<br>(e.g. UCSC)	UCSC hg38

Methodology

Replicates	One sample in each group.
Sequencing depth	The ChIP-seq libraries were prepared using the NEBNext Ultra II DNA Library Prep Kit for Illumina (New England Biolabs), and were run on the HiSeq2000 sequencer (Illumina) to generate single-end 65-bp reads.

Antibodies	Rabbit polyclonal anti-mHSF1j (Akira Nakai Lab Yamaguchi University School of Medicine)<br>Rabbit polyclonal anti-hTRIM24-3 (Akira Nakai Lab Yamaguchi University School of Medicine)<br>Rabbit polyclonal anti-hTRIM33-2 (Akira Nakai Lab Yamaguchi University School of Medicine)
Peak calling parameters	Sequenced reads obtained by performing ChIP-seq were mapped to the human genome (UCSC hg38) using Bowtie version 1.1.2 with "-n2 -m1" option, which allows two mismatches in the first 28 bases per read and outputs the uniquely mapped reads. Redundantly mapped reads (reads starting exactly at the same 5'-sequence ends) were filtered out for further analysis. For peak-calling and data-visualization, we used DROMPA3 version 3.7.2 with default parameter set, which normalizes the read distribution with the total number of mapped reads and identifies the regions that satisfy following criteria. P-value of ChIP read enrichment, $p < 1 \times 10-4$, and P-value of fold enrichment (ChIP/Input), $p < 1 \times 10-4$.
Data quality	See above.
Software	Bowtie version 1.1.2 (Langmead, B. at al, Genome Biol. 10, R25, 2009).<br>DROMPA3 version 3.7.2 (Nakato, R. at al, Genes Cells 18, 589-601, 2013).
